# Automatic Recognition Reading Method of Pointer Meter Based on YOLOv5-MR Model

**DOI:** 10.3390/s23146644

**Published:** 2023-07-24

**Authors:** Le Zou, Kai Wang, Xiaofeng Wang, Jie Zhang, Rui Li, Zhize Wu

**Affiliations:** 1 School of Artificial Intelligence and Big Data, Hefei University, Heifei 230601, China; zoule@mail.ustc.edu.cn (L.Z.);; 2 Institute of Intelligent Machinery, Hefei Institute of Physical Sciences, Chinese Academy of Sciences, Hefei 230031, China

**Keywords:** deep learning, meter reading, object detection, substation patrol

## Abstract

Meter reading is an important part of intelligent inspection, and the current meter reading method based on target detection has problems of low accuracy and large error. In order to improve the accuracy of automatic meter reading, this paper proposes an automatic reading method for pointer-type meters based on the YOLOv5-Meter Reading (YOLOv5-MR) model. Firstly, in order to improve the detection performance of small targets in YOLOv5 framework, a multi-scale target detection layer is added to the YOLOv5 framework, and a set of Anchors is designed based on the lightning rod dial data set; secondly, the loss function and up-sampling method are improved to enhance the model training convergence speed and obtain the optimal up-sampling parameters; Finally, a new external circle fitting method of the dial is proposed, and the dial reading is calculated by the center angle algorithm. The experimental results on the self-built dataset show that the Mean Average Precision (mAP) of the YOLOv5-MR target detection model reaches 79%, which is 3% better than the YOLOv5 model, and outperforms other advanced pointer-type meter reading models.

## 1. Introduction

Pointer-type meters are widely used in power systems, manufacturing systems, military, and aerospace due to their simple structure, low design and manufacturing costs, strong anti-interference ability, and high reliability. The traditional manual method of periodically checking meter readings is not only inefficient and inaccurate, but also cannot provide real-time readings. The traditional method requires a significant amount of human and material resources, and in certain extreme environments, such as those characterized by high temperature, high pressure, and radiation, manual reading of pointer instruments can be inconvenient. Currently, meter reading models are mainly based on traditional image processing techniques, which fail to effectively address challenges such as uneven illumination, complex backgrounds, tilted pointers, and blurred images in each image. As a result, the processing is cumbersome, the accuracy is low, and the error is significant. Thus, there is a pressing need to adopt deep learning technology to develop a meter reading model for pointer-type instruments.

In early research, both domestic and international researchers used a series of traditional image processing techniques to process pointer-type instrument panels. Alegria et al. [1] were the first to use image processing and computer vision techniques to automatically read pointer-type instrument readings for data communication interfaces. In recent years, Yue et al. [2] implemented automatic reading of pointer-type instruments based on machine vision technology, proposing a distance discrimination method based on the distance from the pointer to the adjacent scale line on the left, for scale line and pointer positioning. However, this method required two clearer images for subtraction to extract the pointer, and was not robust against strong external lighting changes and other interferences. Sun et al. [3] proposed using the concentric ring search method to determine the deflection angle of the instrument pointer, which had higher accuracy for reading pointer-type instruments. In order to achieve automatic reading of meters, Belan et al. [4] used a method combining radial projection with the Bresenham algorithm to identify the position of the pointer in the instrument panel, thereby obtaining the meter reading. Liu et al. [5] proposed a machine vision-based an automatic meter reading method that used a region-growing algorithm to locate the center of the dial, and extracted the pointer through contour fitting; it can automatically read instruments with evenly or unevenly distributed scale lines, and has good accuracy. Fang et al. [6] used the SIFT algorithm to match the instrument dial area, and then used algorithms such as the Hough transform to further process the instrument pointer, calculating the deflection angle of the pointer to achieve automatic reading of the pointer-type instrument. Huang et al. [7] proposed an instrument detection algorithm for multiple instrument types, and a single-camera visual pointer reconstruction algorithm to accurately read the scale of the pointer-type instrument. Gao et al. [8] extracted the connected components of the pointer by analyzing the contour of the instrument, and proposed using the support vector machine and histogram gradient method to recognize the numbers on the instrument panel. They then used Newton interpolation linear relationships to determine pointer errors and achieved an automatic reading of the instrument. Ma et al. [9] proposed a method based on symmetric binarization thresholds to segment the pointer region, and an improved random sample consistency algorithm to identify the pointer, which had high adaptability to background interference and pointer shadow issues in complex environments. The research on automatic reading of pointer-type instruments has gradually matured both domestically and internationally, with strong robustness against factors such as lighting and background interference, but most algorithms still have poor robustness and accuracy.

In recent years, with the development of computer technology and artificial intelligence [10,11], automatic meter reading based on deep learning has been widely developed. Currently, most meter reading methods based on deep learning technology are implemented based on object detection methods [12], and deep neural networks have become the main method for object detection. The task of object detection is to find the regions of interest in an image and determine their position, size, and category information. Existing deep learning detection methods can be mainly divided into two categories. One category is the two-stage detection model represented by R-CNN [13], SPP-NET [14], Faster R-CNN [15], and Mask R-CNN [16]. These models have high accuracy but slow detection speed. The other category is the one-stage detection model represented by the YOLO series [17] and SSD [18], which is based on regression. These models have faster computation speeds but lower detection accuracy. In 2016, Redmon et al. proposed the YOLOv1 [17] model, which for the first time regressed the problem of object localization and classification as a whole. The model divided the image into S × S grids and predicted two candidate boxes of different sizes on each grid, and then used non-maximum suppression (NMS) to eliminate duplicates and obtain the final prediction boxes. Although YOLOv1 had a fast detection speed, its detection performance was poor due to the small number and fixed size of predicted boxes on each grid. In 2017, Redmon et al. proposed the YOLOv2 [19] model, which retained the idea of grid division in YOLOv1, and introduced the Anchor concept from Faster R-CNN, greatly increasing the detection accuracy of the model. However, the scale of Anchor in YOLOv2 was relatively single, and its detection performance for multi-scale targets was not very good. In 2018, Redmon et al. [20] proposed the YOLOv3 model, which introduced the feature pyramid and achieved multi-scale object prediction. In 2020, Bochkovskiy et al. proposed the YOLOv4 [21] model, which combined the idea of the cross-stage local network and improved the main network, achieving a dual improvement in detection accuracy and speed. In 2020, the Ultralytics team proposed the YOLOv5 [22] model, which has four versions of different sizes, from small to large: YOLOv5s, YOLOv5m, YOLOv5l, and YOLOv5x. The YOLOv5 models of different sizes have similar structures, and only control the network layers and the number of input and output channels of each layer through two parameters, depth and width, to obtain four models of different sizes. The entire YOLOv5 network can be divided into an input end, a backbone network, a Neck network, and a prediction end. The backbone network is used to extract features, the Neck network is used to fuse features, and the prediction end regresses the predicted results based on the input features. Although YOLOv5 has not proposed a novel model system for the YOLO series, it is a culmination of many optimization strategies and training techniques, achieving the highest performance of YOLO series of object detection algorithms, and providing a very convenient model training and deployment scheme. 

Deep learning is a popular topic in computer vision [23], and studies have investigated recognition reading algorithms for pointer meter. For example, Liu et al. [24] used Faster R-CNN to locate the meter position and employed Hough transform for pointer detection and reading. Similarly, Wang et al. [25] used Faster-RCNN to detect the target meter panel area and proposed a Poisson fusion method to expand the dataset. They used image processing methods for preprocessing and Hough transform for pointer centerline detection. Wu et al. [26] proposed an automatic reading system based on computer vision, addressing issues of poor robustness, high training costs, inadequate compensation correction, and low accuracy. They designed a meter image skew correction algorithm using binary masks and improved Mask-RCNN for different types of pointer meters. Zou et al. [27] used Mask-RCNN for pointer segmentation and achieved high-precision meter reading recognition. Li et al. [28] presented a dial reading algorithm for inspection robots that solved problems of uneven image illumination, complex backgrounds, and interference through techniques like image enhancement, circle detection, pointer detection, and automatic reading. However, most of these algorithms are based on the Faster-RCNN algorithm. YOLOv5 has several advantages over Faster R-CNN. For instance, YOLOv5 has a faster detection speed due to its new network structure, which can process input images more efficiently. Moreover, YOLOv5 has a simpler network structure, making it easier to implement and use. Additionally, YOLOv5 can run directly on a single GPU, while Faster R-CNN requires multiple GPUs to achieve high-speed detection. Furthermore, YOLOv5 has higher detection accuracy on some public datasets than Faster R-CNN, and supports multiple input sizes to adapt to different image sizes. In terms of model deployment, YOLOv5 has smaller model files and can be deployed on low-power devices more easily, while Faster R-CNN requires more custom code for deployment, which may increase deployment difficulty and time. 

In engineering applications of object detection, single-stage object detection models are more widely used than two-stage models due to their high real-time requirements. Among them, the YOLO series of models have become the most user-friendly object detection models with high accuracy, fast speed, and ease of deployment through continuous development [21]. However, the YOLOv5 model still has shortcomings in detecting small objects. The first reason for the poor detection of small objects by the YOLOv5 model is that the sample size of small objects is small, and the down-sampling factor of the YOLOv5 model is relatively large, making it difficult for deeper feature maps to learn the feature information of small objects. The second reason is that the Generalized Intersection over Union (GIoU) [29] loss function used by YOLOv5 for small object detection is slow and unstable in convergence. The third reason is that the YOLOv5 model uses the nearest-neighbor interpolation method for up-sampling, which requires manually designed up-sampling parameters and is difficult to obtain optimal up-sampling parameters.

In this paper, a new pointer automatic meter reading model, named YOLOv5-MR, is proposed and combined with a new method of fitting the dial outer circle to improve the accuracy of meter reading. Compared with the original YOLOv5 model, YOLOv5-MR adds detection to the $C_{2}$ feature layer, which can extract more target features, especially when improving the detection of small objects. In order to solve the problem of edge length error amplification in the convergence process of the model, this paper uses the Efficient Intersection over Union (EIoU) [30] loss function to improve the original GIoU loss function, which makes the model convergence more stable. Meanwhile, in order to learn better up-sampling parameters, this paper adopts the transposed convolution method instead of the fixed nearest neighbor interpolation method. Finally, this paper proposes a new method to fit the outer circle of the dial to calculate the meter readings using the circular angle algorithm. After experimental validation, the model performs well in small object detection, while the introduced penalty term helps to avoid erroneous length amplification, thus achieving faster and better model convergence. In addition, the proposed method of fitting the dial outer circle outperforms existing object detection-based meter reading methods in terms of accuracy, speed and robustness. We also present a new dataset for lightning arrester meter reading named MR-Meter.

The paper is organized as follows. Section 2 presents related work in the field of automatic meter reading. Section 3 describes the proposed YOLOv5-MR model, including the improvement of the original YOLOv5 model and a new method for fitting the dial outer circle. Section 4 presents the experimental results and analysis. Finally, Section 5 discusses the advantages of the model proposed in this paper over other models. Section 6 summarizes the paper and discusses future work.

## 2. Related Works 

### 2.1. Object Detection

Deep object detection models can be categorized into two types: two-stage detectors and one-stage detectors. Faster R-CNN [15] is a classic two-stage deep learning model that uses a Region Proposal Network (RPN) to propose candidate object regions and performs classification and refinement on these proposals. However, for small objects like watch dials, it is difficult to match the size of the candidate regions with the size of the objects, leading to inaccurate detection results.

One-stage detectors, YOLO [17,19,20,21] and SSD [18], are based on single-stage detection, and YOLO is simpler and easier to train and adjust, compared to SSD. To improve the detection and recognition rates of YOLOv2 [19], YOLOv3 [20] also introduces a concept called multi-scale prediction, where the model predicts objects at three different scales, allowing it to detect small objects more accurately. YOLOv4 [21] builds upon the success of previous YOLO versions and introduces various improvements such as a new backbone network, a better neck network, and a new loss function. These improvements led to state-of-the-art performance on the COCO dataset. YOLOv5 [22], on the other hand, is a completely new architecture that uses a novel approach to object detection.

### 2.2. YOLOv5 Network Architecture

**Figure 1:**
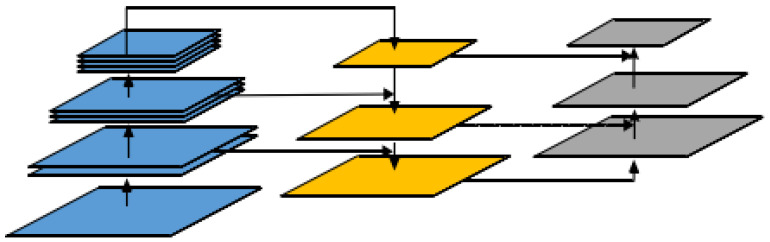
PANet architecture.

**Figure 2:**
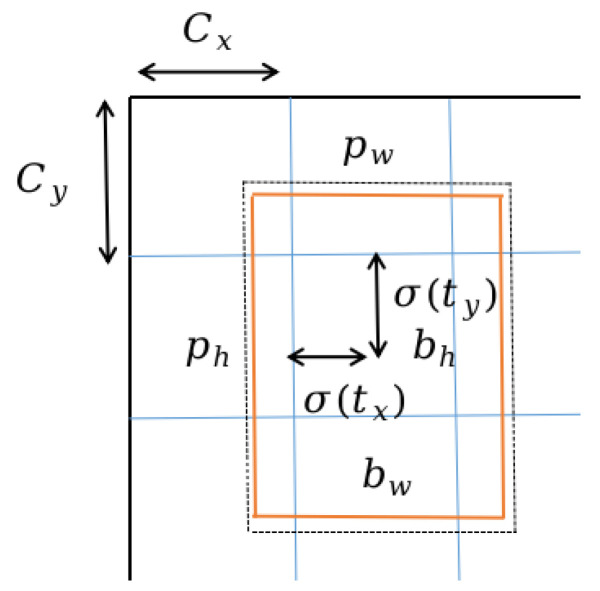
Illustration of the predicted bounding box.

In the YOLOv5 model, the Neck network is responsible for feature enhancement by processing the features extracted from the backbone network to improve the accuracy of predictions. The original Feature Pyramid Network (FPN) structure used in YOLOv5’s Neck network employs a top-down feature fusion method to address the multi-scale variation problem in object detection, which has been widely used in many models. However, if only the FPN structure is used to fuse contextual information, communication between upper-level and lower-level information cannot be achieved. Therefore, the YOLOv5 model adds a Path Aggregation Network (PAN) structure on top of the FPN to introduce a bottom-up information flow, which fully integrates the top-down and bottom-up information flows to enhance the detection ability of the network. This results in the PANet network, which has been shown to improve performance in object detection tasks. The PANet network structure is shown in Figure 1:

The bottom-up structure enhances the information transfer between different feature maps by fusing shallow features with rich position information to deeper features. This method accurately preserves spatial information and effectively improves the detection capability of the network for large and medium-sized objects. As shown in Figure 2, in the YOLOv5 model, the image is first divided into S × S grids, and the center coordinates of each grid are denoted as $C_{x}$ and $C_{y}$, and the width and height are denoted as $t_{w}$ and $t_{h}$, respectively. In each grid, the corresponding predicted values for the center coordinates (σ($t_{x}$), σ($t_{y}$)) and relative width $t_{w}$ and height $t_{h}$ are output, and the final predicted box (corresponding to the orange solid line) is obtained based on the actual position, width, and height of the grid. The dashed box in Figure 2 represents the prior box, where $p_{w}$ and $p_{h}$ are the width and height of the prior box. For example, in the grid located at the second row and second column in Figure 2, the center point position ($b_{x},{\;b}_{y}$) and width ($b_{w}$) and height ($b_{h}$) of the predicted box are obtained based on Equation (1).

(1) $$b_{x}=2\times \sigma \left(t_{x}\right)-0.5+c_{x}\;\begin{array}{c} \;\;b_{y}=2\times \sigma \left(t_{y}\right)-0.5+c_{y} \end{array}\;b_{w}=p_{w}{\left(\sigma \left(t_{w}\right)\times 2\right)}^{2}\;\;{\;b}_{h}=p_{h}{\left(\sigma \left(t_{h}\right)\times 2\right)}^{2}$$

The final values used in the calculation of the loss function include the width, height, and center point position of the predicted box, the confidence score of the predicted box, and the classification information. The confidence score of the predicted box refers to the Intersection over Union (IoU) between the predicted box and the annotated box. Generally, predicted boxes with IoU > 0.7 are considered positive examples, meaning successfully predicted targets, while those with IoU < 0.3 are considered negative examples, meaning background. Ignoring other predicted boxes, the loss function is calculated using positive and negative examples, while attempting to maintain a balance between the numbers of positive and negative examples. The classification information refers to the probability that the predicted box contains a target of a certain category. The IoU is shown in Equation (2). The values used in the computation of the loss function for object detection include the width, height, and center point position of the predicted box, the confidence score of the predicted box, and the classification information. The confidence score of the predicted box is defined as the IoU between the predicted box and the ground truth box. Generally, a predicted box with an IoU greater than 0.7 is considered a positive example, meaning that the target object has been successfully detected. Objects with an IoU less than 0.3 are considered negative examples, representing the background. The loss function is calculated using only positive and negative examples while striving to maintain a balance between the two classes. The classification information refers to the probability that the predicted box contains an object of a certain category. The IoU is defined as shown in Equation (2), where ${\mathrm{Box}}_{1}$ represents the predicted box and ${\mathrm{Box}}_{2}\;$ represents the ground truth box.

(2) $$\;\mathrm{IoU}=\frac{{\mathrm{Box}}_{1}\cap {\mathrm{Box}}_{2}}{{\mathrm{Box}}_{1}\cup {\mathrm{Box}}_{2}}$$

The YOLOv5 model’s loss function consists of three components: classification loss, localization loss, and object confidence loss. YOLOv5 employs the GIoU [29] as the bounding box regression loss function. The GIoU method overcomes the shortcomings of IoU while fully utilizing its advantages. Let A be the predicted box and B be the ground truth box, and let $A_{c}$ represent the minimum convex hull containing both A and B. The GIoU loss function is calculated as shown in Equation (3):

(3) $$\mathrm{GIoU}=\mathrm{IoU}-\frac{A_{c}-U}{A_{c}}$$

During the training phase, binary cross-entropy (BCE) loss is used as the classification loss. Therefore, the complete loss function consists of three components: bounding box regression loss (first term), object confidence prediction loss (second and third terms), and class prediction loss (fourth term). The loss function is formulated as shown in Equation (4):

(4) $$\begin{array}{c} \mathrm{Loss}(\mathrm{obj})=\;\mathrm{GloUloss}\;\\ +\sum_{i=0}^{S\times S}\sum_{j=0}^{B}1_{\mathrm{ij}}^{\mathrm{obj}}\left[C_{i}\log\left(C_{i}\right)+\left(1-C_{i}\right)\log\left(1-C_{i}\right)\right]\\ +\sum_{i=0}^{S\times S}\sum_{j=0}^{B}1_{\mathrm{ij}}^{\mathrm{obj}}\left[C_{i}\log\left(C_{i}\right)+\left(1-C_{i}\right)\log\left(1-C_{i}\right)\right]\\ -\sum_{i=0}^{S\times S}\sum_{j=0}^{B}1_{\mathrm{ij}}^{\mathrm{noobj}}\left[C_{i}\log\left(C_{i}\right)+\left(1-C_{i}\right)\log\left(1-C_{i}\right)\right]\\ -\sum_{i=0}^{S\times S}\sum_{j=0}^{B}1_{\mathrm{ij}}^{\mathrm{noobj}}\left[C_{i}\log\left(C_{i}\right)+\left(1-C_{i}\right)\log\left(1-C_{i}\right)\right] \end{array}$$

In Equation (4), S is the grid scaling factor, B is the number of predicted boxes per grid cell, C is the total number of classes, p is the class probability, and $x_{i},{\;y}_{i},{\;w}_{i},{\;h}_{i}$ represent the center point coordinates, width, and height of the predicted box in the ith grid cell. The weight coefficient for the bounding box coordinates is denoted as $\lambda_{\mathrm{coord}}$, and the penalty coefficient for the objectness prediction is denoted as $\lambda_{\mathrm{noobj}}$.

In order to solve low precision and large error of the current meter reading, we present a novel pointer-type automatic meter reading model based on YOLOv5, which aims to address the significant challenges of low accuracy and large errors in current object detection-based meter reading methods.

## 3. YOLOv5-MR Network Architecture

### 3.1. Multi-Scale Feature Detection

**Figure 3:**
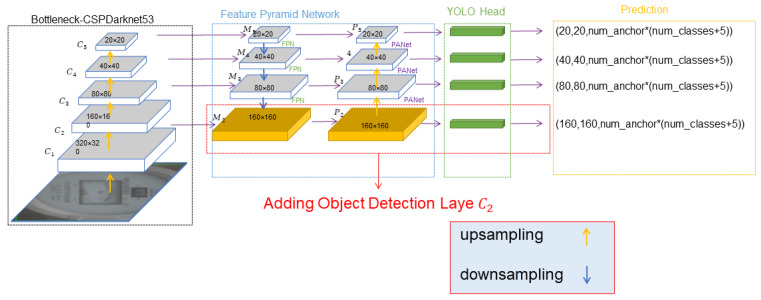
YOLOv5-MR network structure.

To improve the detection ability of the model for small targets and detect more target features, this paper proposes a new object detection model, YOLOv5-MR. The overall structure of the YOLOv5-MR model is shown in Figure 3. The model is divided into the input layer, feature extraction layer, feature fusion layer, and detection head. To enable YOLOv5 to detect more small target features, we add the $C_{2}$ target detection layer to detect more shallow features. At the same time, a set of custom anchors are used on the $C_{2}$ target detection layer to better converge and fit small targets in the dataset. Secondly, the EIoU loss function is used to make the model converge more stably and solve the problem of error side length enlargement that may exist during the convergence process. Finally, the paper uses transpose convolution to learn better up-sampling parameters.

In the field of computer vision, detecting small objects has long been a challenge in object detection, aiming to accurately detect objects with very few visual features (objects with a size of 32 pixels × 32 pixels or smaller) in an image. In the deep feature extraction process of neural networks based on the basic unit of CNN, the information on the feature map decreases as the network deepens, through continuous convolution and pooling operations. For example, Faster R-CNN use VGG16 as the backbone network, after the last convolution operation, the height and width of the feature map become one-sixteenth of the original image. Therefore, with continuous convolution, the information of small objects on the feature map is constantly decreasing, which leads to the failure to detect some small objects, and subsequently, the failure in classification and bounding box regression.

After continuous convolution and pooling operations, the information on the feature map is constantly decreasing. In Figure 3, the $C_{2}$ feature layer contains more object information than the $C_{3}$ feature layer. Therefore, this paper adds detection on the $C_{2}$ feature layer, which contains more feature information. In the original YOLOv5 algorithm, YOLOv5 uses the FPN original model, which only has three detection layers and corresponds to three sets of initialized Anchor values. When the input image size is 640 × 640, the detection layer size corresponding to $C_{3}$ is 80 × 80, which can be used to detect objects larger than 8 × 8; the detection layer size corresponding to $C_{4}\;$ is 40 × 40, which can be used to detect objects larger than 16 × 16; the detection layer size corresponding to $C_{5}$ is 20 × 20, which can be used to detect objects larger than 32 × 32. To improve the model’s ability to detect small objects; after the $M_{3}$ layer, the feature map continues to be up-sampled and processed to further expand the feature map. At the same time, the obtained feature map with a size of 160 × 160 is concatenated with the $C_{2}$ feature layer in the backbone network for feature fusion, in order to obtain a larger feature map for small object detection. Therefore, this paper adds the $P_{2}$ detection layer in the feature fusion part, using four feature layers ($P_{2}$ $P_{3}$ $P_{4}$ $P_{5})$ for detection.

The original YOLOv5 detection model uses default anchor boxes designed for the COCO dataset, which generate nine anchors of different scales and aspect ratios to detect small objects on large feature maps. The sizes of the anchors are (10 13 16 30 33 23), (30 61 62 45 59 119) and (116 90 156 198 373 326).

However, the anchor sizes in this set are designed based on the object sizes in the COCO dataset and are not suitable for the MR-Meter dataset used in this paper. The default anchor sizes in the network cannot converge well to fit small objects in the dataset. Therefore, in consideration of the size characteristics of the meter objects in the MR-Meter dataset, a new set of anchors with sizes of (5, 6, 8, 14, 15, 11) (smaller than the default settings) was added to the network to detect small objects in the meter. During feature extraction, larger feature maps contain more information about small objects. Thus, smaller values are typically assigned to anchor sizes in larger feature maps, while larger values are assigned to anchor sizes in smaller feature maps to detect larger objects. Additionally, non-maximum suppression is used to eliminate redundant candidate regions. The new small-size anchors are used in the newly added large-scale object detection layer $C_{2}\;$ in Figure 3 to detect small objects in the $C_{2}$ feature map. Therefore, during training, four sets of 12 anchors are used, located in the $C_{2}$, $C_{3}$, $C_{4}$, and $C_{5}$ object detection layers, with sizes of: (5 6 8 14 15 11), (10 13 16 30 33 23), (30 61 62 45 59 119) and (116 90 156 198 373 326).

### 3.2. Loss Function

The YOLOv5 model adopts the GIoU loss function [29] for bounding box regression. Although the GIoU loss function addresses the gradient vanishing problem of the IoU loss function, it is unstable and converges slowly. Therefore, this paper use the EIoU loss function [30] for bounding box regression.

The EIoU loss function is an improvement upon the Complete Intersection over Union (CIoU) loss function [29], which itself is an improvement upon GIoU. CIoU is defined by Equation (5):

(5) $$L_{\mathrm{CIoU}}=1-\mathrm{IoU}+\frac{p^{2}\left(b,b^{\mathrm{gt}}\right)}{c^{2}}+\alpha V\;$$

In Equation (5), c is the diagonal length of the box enclosing the two bounding boxes, b and $b^{\mathrm{gt}}$ represent the centers of the predicted and ground-truth bounding boxes respectively, p(.) represents the Euclidean distance between the centers of the predicted and ground-truth boxes, and α is a weight coefficient. V is defined by Equation (6):

(6) $$V=\frac{4}{\pi^{2}}{\left(\arctan\frac{w^{\mathrm{gt}}}{h^{\mathrm{gt}}}-\arctan\frac{w}{h}\right)}^{2}$$

(7) $$\frac{V}{w}=\frac{8}{\pi^{2}}\left(\arctan\frac{w^{\mathrm{gt}}}{h^{\mathrm{gt}}}-\arctan\frac{w}{h}\right)\frac{h}{w^{2}+h^{2}}$$

(8) $$\frac{v}{h}=-\frac{8}{\pi^{2}}\left(\arctan\frac{w^{\mathrm{gt}}}{h^{\mathrm{gt}}}-\arctan\frac{w}{h}\right)\frac{h}{w^{2}+h^{2}}$$

**Figure 4:**
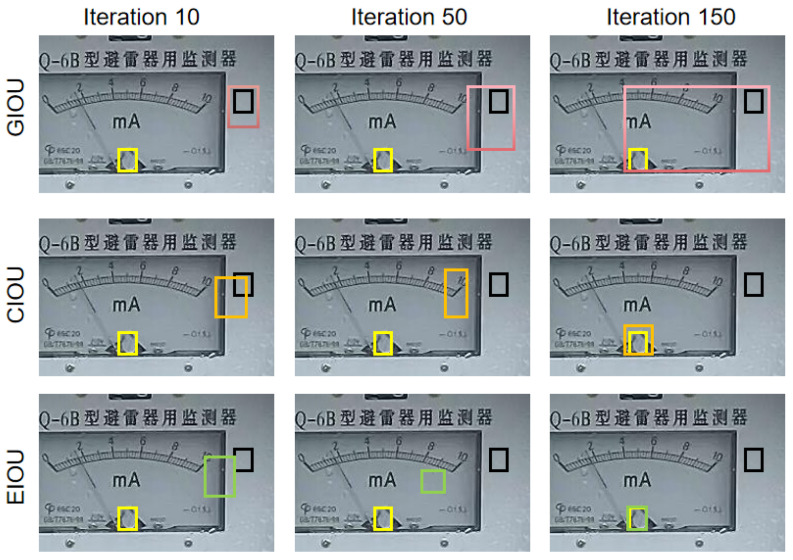
The convergence of the three loss functions of GIoU, CIoU, EIoU at the same anchor point and ground truth, where yellow box represents Ground Truth, black box represents the initial position. The first row represents the convergence process of GIoU, the second row represents the CIoU convergence process, and the third row represents the convergence process of EIoU. The pink box, the brown box and the green box represent the convergence process of the three prediction box from the 10th iteration to the 150th iteration, respectly.

There are two problems with CIoU. Firstly, CIoU uses the relative proportion of width and height instead of their actual values. According to the definition of v, if the predicted width and height satisfy $w={\mathrm{kw}}^{\mathrm{gt}},\;h={\mathrm{kh}}^{\mathrm{gt}}|k\epsilon R^{+}$, then the penalty term added in CIoU for the relative proportion will not be effective. Secondly, from Equations (7) and (8), we can derive that $\frac{v}{w}=-\frac{h}{w}\frac{v}{h},\;$ indicating that the gradients $\frac{v}{w}$ and $\frac{v}{h}$ for width and height have opposite signs. This opposite sign poses a problem during the training process. When one value of width or height increases, the other value must decrease, i.e., the two values cannot increase or decrease together, which affects the convergence speed of the model. As shown in Figure 4, GIoU uses the closure of the area minus the union of the area as a penalty term, which leads to the problem of taking a detour to first expand the union area and then optimize IoU, as shown in the first row of Figure 4. Additionally, in CIoU, the width and height cannot increase or decrease simultaneously. This is demonstrated in the second row of Figure 4. From the Figure 4, it can be seen that EIoU has the best convergence effect.

Anchor is a bounding box whose width and height are both larger than the object to be detected, but it still amplifies the width of the predicted box during optimization. Compared to the two loss functions above, EIoU has a faster convergence speed. Based on this phenomenon, EIoU proposes a loss function that directly penalizes the predicted results of w and h, that is, Equation (9):

(9) $${\;L}_{\mathrm{EIoU}}=1-\mathrm{IOU}+\frac{p^{2}\left(b,b^{\mathrm{gt}}\right)}{C^{2}}+\frac{p^{2}\left(w,w^{\mathrm{gt}}\right)}{C_{w}^{2}}+\frac{p^{2}\left(h,h^{\mathrm{gt}}\right)}{C_{h}^{2}}$$

In Equation (9), C is the diagonal length of the box surrounding the two bounding boxes, $C_{w}\;$ represents the width of the bounding box, $C_{h}$ is the height of the bounding box, b and $b^{\mathrm{gt}}$, respectively, represent the center of the prediction bounding box and the ground live bounding box, w and $w^{\mathrm{gt}}$, respectively, represent the width of the prediction bounding box and the width of the real bounding box, h and $h^{\mathrm{gt}}$, respectively, represent the height of the prediction bounding box and the height of the real bounding box, and p (.) represents the Euclidean distance between the centers of the prediction bounding box and the ground live bounding box.

### 3.3. Transposed Convolution

**Figure 5:**
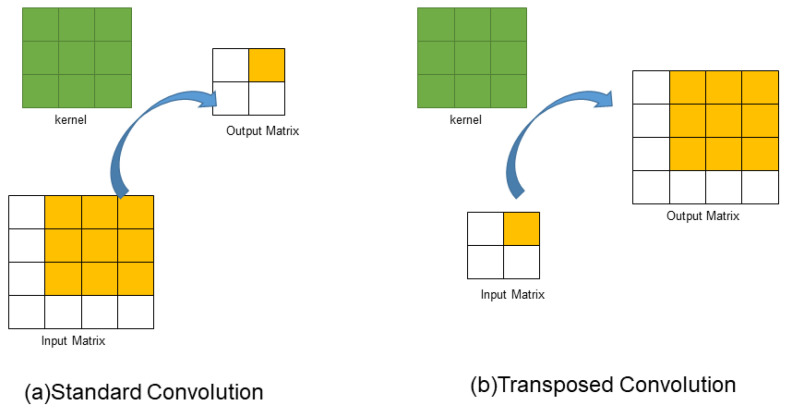
Schematic diagram of (**a**) ordinary convolution and (**b**) transposed convolution.

In the process of using neural networks, it is often necessary to use up-sampling to increase the resolution of low-resolution images. Up-sampling methods include nearest-neighbor interpolation [31], bilinear interpolation [32], and bi-cubic interpolation [33]. These up-sampling methods are based on prior knowledge and have fixed and non-learnable rules, which are not ideal in many scenarios. Therefore, we introduce transposed convolution [34] to learn better up-sampling parameters. Compared with traditional up-sampling methods, the up-sampling method of transposed convolution is not a preset interpolation method, but like standard convolution, it has learnable parameters and can obtain the optimal up-sampling method through model learning. The operation of standard convolution is to multiply the elements in the convolution kernel with the corresponding elements in the input matrix pixel by pixel and sum them up. Then, the convolution kernel slides on the input matrix in units of stride until all positions of the input matrix are traversed. Assuming that the input is a 4 × 4 matrix, a 3 × 3 standard convolution is used for calculation, and padding = 0 and stride = 1. The final output result is a 2 × 2 matrix. Let x be the 4 × 4 input matrix, y be the 2 × 2 output matrix, and C be the 3 × 3 convolution kernel, then the standard convolution process is $C_{x}$ = y. The process of standard convolution is shown in (a) of Figure 5. Figure 5b shows the transposed convolution schematic.

The main purpose of transpose convolution [34] is to perform up-sampling. Transpose convolution is not the inverse operation of convolution, and it can only restore the size of the feature map to its original size, but the data is different from the original. Transpose convolution does not increase the receptive field size, it only learns the content of a feature point and then maps it to the convolution kernel. Therefore, the receptive field of all points on the convolution kernel remains unchanged, and adding multiple layers of convolution behind it can expand the receptive field range. Using transpose convolution for up-sampling in the model can automatically learn better up-sampling parameters and achieve higher accuracy. Therefore, in the feature extraction part of the YOLOv5-MR model, transpose convolution is used to improve the up-sampling ability.

### 3.4. Recognition and Reading of Dial Numbers

**Figure 6:**
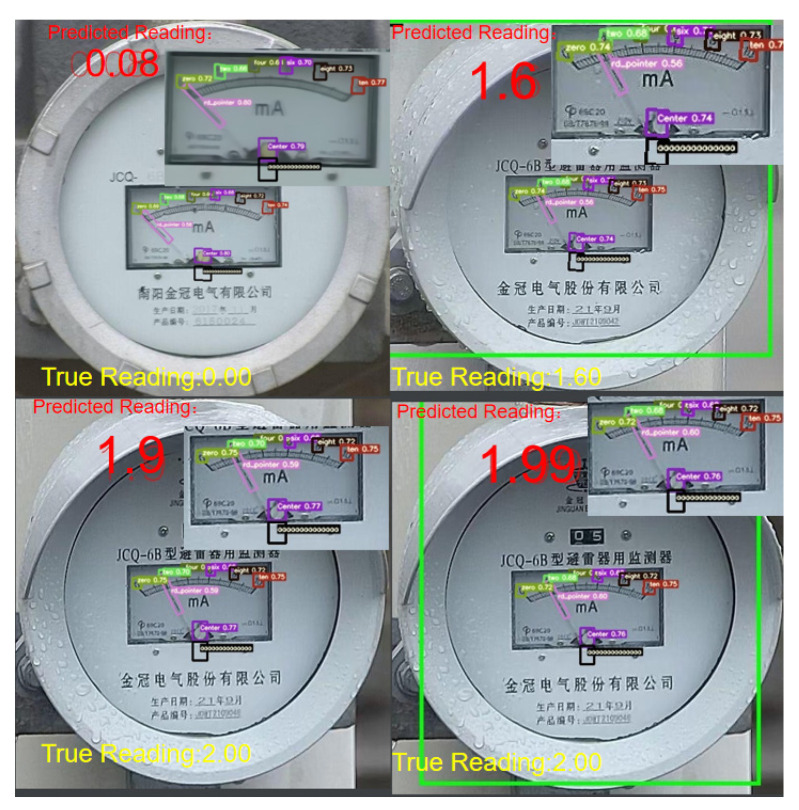
The recognition effect of the YOLOv5-MR model. Predicted-Reading represents the predicted reading, and True Reading represents the actual reading. In the rectangular box in the center of the index dial, the green number 2 represents the scale value 2, the yellow number 0 and 4 represents the scale value 0 and 4, the purple number 6 represents the scale value 6, the black number 8 represents the scale value 8, and the red number 10 represents the scale value 10. The purple center represents the predicted dial center, and the black 000… 0 represents the true center of the dial.

The final scale value of the dial needs to be calculated using the coordinates of the pointer, the center point of the dial, and the coordinates of six scale targets. Firstly, the YOLOv5-MR model is trained to detect eight types of targets, including the pointer, the center point of the dial, and six scale values (0, 2, 4, 6, 8, 10). After training, the model is used to locate the corresponding candidate regions in the original image, as shown in Figure 6.

**Figure 7:**
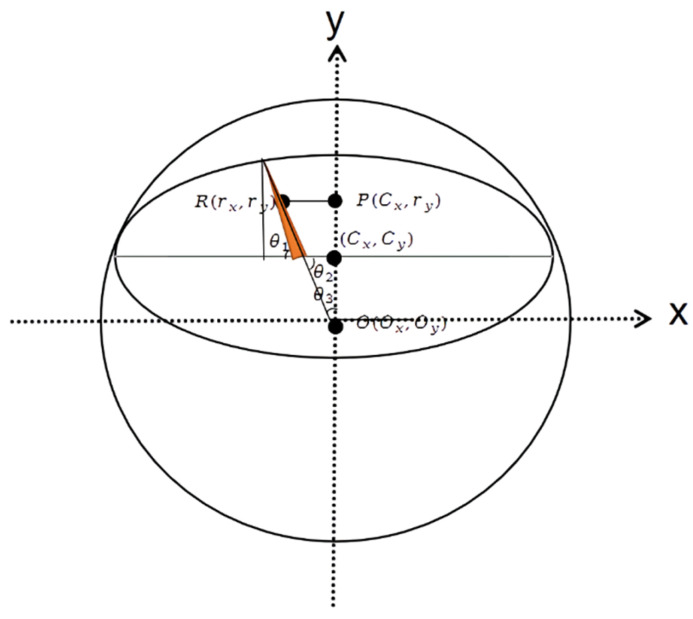
Schematic diagram of the smallest circumscribed circle fitted to an ellipse.

The YOLOv5-MR model obtains the center points of the scale $C(C_{x},C_{y}$), the pointer $R(R_{x},R_{y}$), and the center point of the scale $N_{i}$ ($N_{\mathrm{ix}}$, $N_{\mathrm{iy}}$). However, the predicted center points are not the center of the circle but the center of an ellipse. Directly calculating using Equations (6)–(10) would result in large errors, so it is necessary to fit the circumscribed circle of the ellipse. The schematic diagram of fitting the minimum circumscribed circle of the elliptical dial is shown in Figure 7.

In Figure 7, C($C_{x}$, $C_{y}$) represents the center point of the ellipse-shaped dial predicted by the YOLOv5-MR rotation object detection model, while R($r_{x},r_{y}$) represents the center of the predicted pointer. $P(C_{x},C_{y}$) is a point with the same horizontal coordinate as the center of the dial and the same vertical coordinate as the center of the pointer. $O(O_{x},{\;O}_{y}$) is the center of the finally predicted circumscribed circle. Since the YOLOv5-MR rotation object detection model is used, the rotation angle of the pointer can be directly predicted by the model and denoted as ${\;\theta }_{i}$. According to the principle of equal opposite angles, it is known that $\theta_{1}\;$=${\;\theta }_{2}$ and $\theta_{3}=90-{\;\theta }_{2}$. The length of line RP is denoted as $l_{x}\;$=${\;C}_{x}\;$−${\;r}_{x}$, and $l_{\mathrm{PO}}={\;l}_{\mathrm{RP}}$ is obtained by tanθ =${\;l}_{\mathrm{RP}}$. Finally, the coordinates of the center of the circumscribed circle are $O_{x}={\;O}_{y}$ and $O_{y}\;$=${\;r}_{y}$+$l_{\mathrm{PO}}$. When calculating the reading value based on the predicted center point position, pointer center point position, and dial scale position information, it is necessary to check whether the detection box is complete. The conditions for calculating the reading result are: (1) center point; (2) pointer; and (3) at least two scale marks. If these conditions are not satisfied, the dial scale prediction cannot be performed normally.

**Figure 8:**
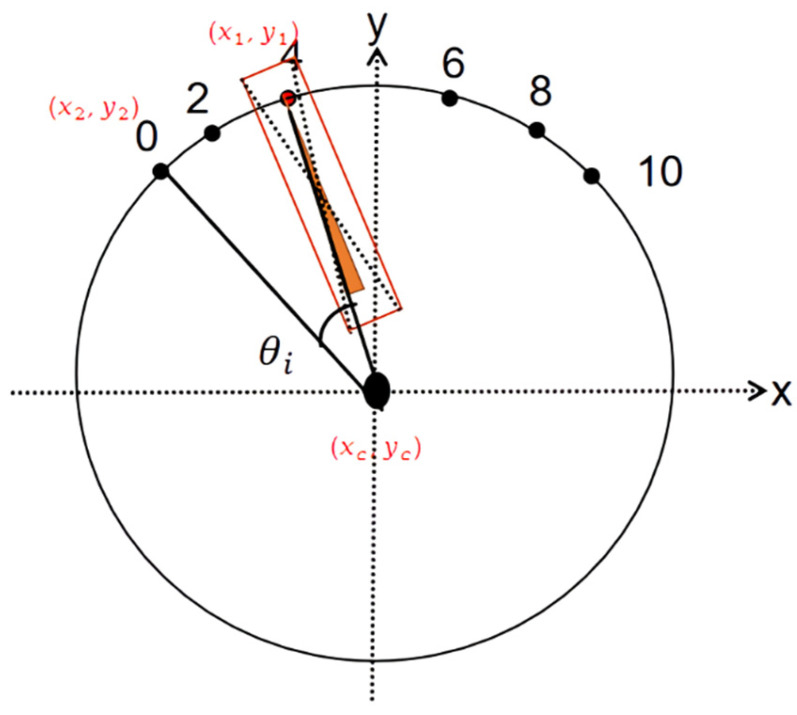
Calculates the angle between the individual tick marks of the pointer.

The process for calculating the angle between the pointer and each scale on the dial is shown in Figure 8, where 0, 2, 4, 6, 8, and 10 denote the dial scales, $(x_{c},{\;y}_{c}$) represents the center of the minimum enclosing circle, ($x_{1},{\;y}_{1})\;$ represents the center of the pointer, and ($x_{2}$, $y_{2}$) represents the coordinates of the 0-scale. The angle between the pointer and each scale, denoted by $\theta_{i}$ (I = 1,2…6), is obtained by Equation (10). This angle calculation method calculates the angle required for the pointer to rotate counterclockwise or clockwise from ($x_{1},{\;y}_{1}$)to the current scale with ($x_{c},{\;y}_{c})\;$ as the rotation center. Counterclockwise angles are negative, and clockwise angles are positive. θ ∈ (−180, 180) forms an angle vector V = [$\theta_{1}$ $\theta_{2}$ $\theta_{3}$ $\theta_{4}$ $\theta_{5}$ $\theta_{6}$].

The mathematical principle for calculating the angle between two vectors based on their coordinates is as follows: Let m and n be two non-zero vectors, and let <m,n> denote the angle between them. Then, the cosine of <m,n> is given by $\cos<m,n\geq \frac{m.n}{\left|m\right|\left|n\right|}$. If the vectors are represented by their coordinates and the dot product operation is used, then we can obtain:

(10) $$\;m=\left(x_{1},y_{1},z_{1}\right),n=\left(x_{2},y_{2},z_{2}\right)$$

(11) $$m.n=\left(x_{1}x_{2}+y_{1}y_{2}+z_{1}z_{2}\right)$$

(12) $$\left|m\right|=\sqrt{x_{1}^{2}+y_{1}^{2}+z_{1}^{2}}$$

(13) $$\left|n\right|=\sqrt{x_{2}^{2}+y_{2}^{2}+z_{2}^{2}}$$

By substituting Equations (10)–(13) into Equation (14), the cosine value of the angle between the two vectors can be obtained, and thus the degree of the vector angle can be obtained.

(14) $$\;\;\cos<m,n>=\frac{\left(x_{1}x_{2}+y_{1}y_{2}+z_{1}z_{2}\right)}{\sqrt{x_{1}^{2}+y_{1}^{2}+z_{1}^{2}}\times \sqrt{x_{2}^{2}+y_{2}^{2}+z_{2}^{2}}}$$

Setting z = 0 in Equation (14), it yields a two-dimensional plane vector, as shown in Equation (15):

(15) $$\cos<m,n>=\frac{\left(x_{1}x_{2}+y_{1}y_{2}\right)}{\sqrt{x_{1}^{2}+y_{1}^{2}}\times \sqrt{x_{2}^{2}+y_{2}^{2}}}$$

The range of the angle between two vectors is $\theta \in \left[0,\pi \right]$. If the scale to be read is located on the left side of the pointer, it is rotated counterclockwise, and the angle value is negative. The larger the negative value, the closer the scale is to the pointer. Similarly, if the scale to be read is on the right side of the pointer, it is rotated clockwise, and the angle value is positive. The smaller the positive value, the closer the scale is to the pointer. After obtaining the angles between each scale and the pointer, the smallest positive angle $\theta_{i}$ and the largest negative angle $\theta_{j}$ are determined. Based on the proportions of these two angles in the entire angle (360 degrees) of the circle, the scale value that the pointer points to is determined. The proposed method directly uses the recognized pointer coordinates, scale coordinates, and fitted center point coordinates for calculation, which is simpler and more convenient than the method proposed by Zou et al. [27], which first extracts the pointer from the image using Mask R-CNN, and then uses the Hough transform to fit the pointer line and obtain the pointer rotation angle.

## 4. Experimental and Results Analysis

### 4.1. Recognition and Reading of Dial Numbers

In this paper, the hardware environment used to conduct the experiments in this paper consisted of an NVIDIA GTX3090 GPU, an Intel(R) Core (TM) i7 CPU with a clock speed of 2.3~4.6 GHz, and 32 GB of RAM. The algorithms were implemented using Pytorch 3.6, Python 3.9, and cuda 10.0, and the experiments were conducted on an ubuntu 18.4 operating system.

All models were trained, validated, and tested under the same hyper-parameters. The hyper-parameters are set as shown in Table 1. The evaluation of the performance of all detections is based on the IOU (Intersection over Union) between the predicted and ground truth bounding boxes. A predicted bounding box is considered successful if its IoU is greater than 0.5. In object detection, both precision and recall need to be considered when evaluating the performance of a network model. The mAP is generally used to evaluate the performance of network models in object detection. The precision and recall are calculated by the Formulas (16) and (17), respectively. The parameter values presented in Table 1 are dimensionless and do not have specific units of measurement.

(16) $$\mathrm{Precision}=\frac{\mathrm{TP}}{\mathrm{TP}+\mathrm{FP}}$$

(17) $$\mathrm{Recall}=\frac{\mathrm{TP}}{\mathrm{TP}+\mathrm{FN}}$$

In the context of object detection, TP refers to the number of true positive detections, FP refers to the number of false positive detections, and FN refers to the number of false negative detections. Average Precision (AP) is defined as the average precision at different recall levels, and is commonly used to evaluate the detection performance of a specific class. mAP is the mean of AP across all object classes, and is commonly used to evaluate the overall performance of a detection model. The calculation of mAP can be expressed by Equation (18).

(18) $$\mathrm{mAP}=\frac{\sum_{i=1}^{n}{\mathrm{AP}}_{i\;}}{n}$$

The ${\mathrm{AP}}_{i}\;$ represents the detection accuracy of a certain class, and n is the number of classes.

### 4.2. Dataset

As there is no publicly available dataset for lightning arrester meter reading, this paper presents a new dataset named MR-Meter, which consists of 2000 lightning arrester images and 14,000 annotated targets. The images were mainly collected from a well-known power station in the region and were uniformly resized to 640 × 640. The dataset was annotated in the VOC2007 format, and the annotation includes the rd_pointer, which refers to the position of the pointer, the Center refers to the center of the pointer, and the positions of the zero, two, four, six, eight, and ten scales. During annotation, efforts were made to ensure that the center of the annotation box is on the scale as much as possible. The training and testing sets were randomly sampled at a ratio of 7:3, with 1400 images for training and 600 images for testing. All annotation information, including location and class information, was recorded in xml format.

**Figure 9:**
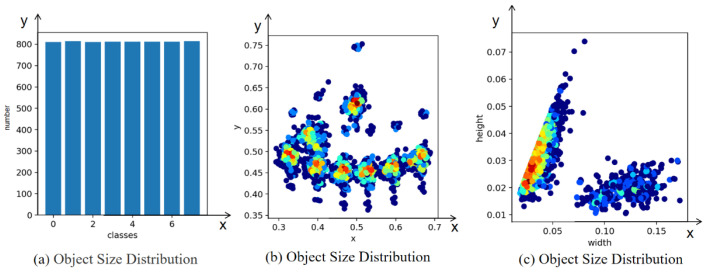
Setting the cluster center to 8 for k-means clustering.

In order to obtain better prior knowledge of the dial targets, this paper uses the K-means algorithm to generate eight prior boxes on the MR-Meter dataset. The clustering results are shown in Figure 9, where (a) represents clustering the target sizes in the dataset, (b) represents clustering the target center positions in the dataset, and (c) represents the statistics of the number of labels in the dataset. From Figure 9, it can be seen that many points are clustered in the lower left corner, and many target sizes are smaller than 0.04, indicating that there are many small targets in the dial dataset.

### 4.3. Results of the YOLOv5-MR Reading Model

This section employs comparative experiments to verify the effectiveness of the YOLOv5-MR model, using the average error δ as the evaluation metric, as shown in Equation (19):

(19) $$\delta =\frac{\sum_{n}^{i=1}\frac{\left|\alpha_{i}-A_{i}\right|}{A_{i}}}{n}$$

where n denotes the number of experimental groups, $a_{i}$ represents the predicted value of the model, and $A_{i}\;$ represents the manual reading. We used 10 sets of data, and the recognition results are shown in Table 2.

**Figure 10:**
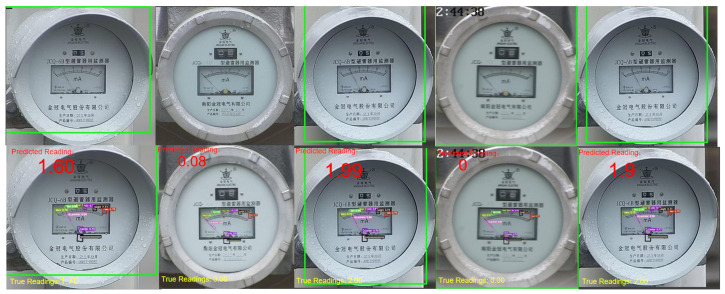
The first row represents the original graph, and the second row represents the YOLOv5-MR recognition results. Predicted-Reading represents the final reading result of the YOLOv5-MR model, and True Reading represents the actual reading. In the rectangular box in the center of the index dial of the second row, the green number 2 represents the scale value 2, the yellow number 0 and 4 represents the scale value 0 and 4, the purple number 6 represents the scale value 6, the black number 8 represents the scale value 8, and the red number 10 represents the scale value 10. The purple center represents the predicted dial center, and the black 000… 0 represents the true center of the dial.

According to the data in Table 2, it can be seen that YOLOv5-MR can effectively improve the detection performance of small targets in the dataset, and has strong convergence ability. Therefore, the prediction performance of YOLOv5-MR model has a smaller error compared to YOLO series object detection models. The final recognition results of YOLOv5-MR are shown in Figure 10.

### 4.4. Experimental Results

**Figure 11:**
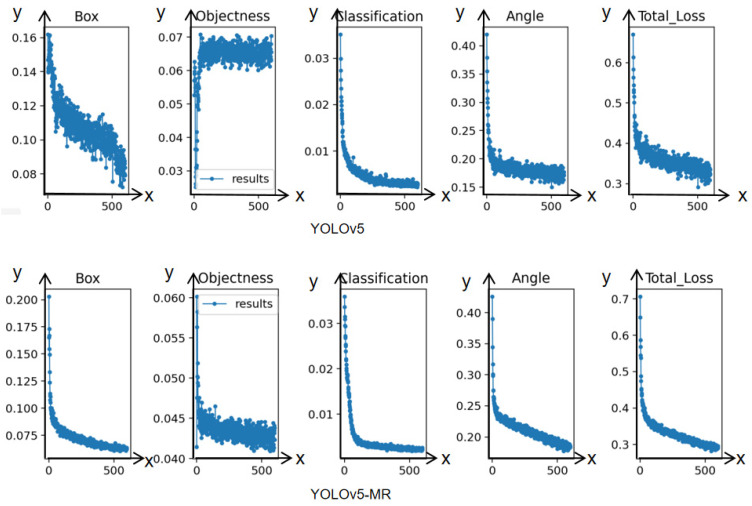
Loss comparison chart.

To compare the performance of our YOLOv5-MR model with the YOLOv5 model, we conducted experiments using the same dataset and parameter settings. We plotted the loss curves of the two models based on the saved log files during training, as shown in Figure 11. Each image from left to right represents the Box_Loss, Obj_Loss, Cls_Loss, Ang_Loss, and Total_Loss, respectively. From the Total_Loss curves, we can see that the YOLOv5-MR model converges faster, more stably, and with a smaller loss value than the YOLOv5 model. This indicates that the EIoU loss function, which directly penalizes the predicted box’s aspect ratio, can effectively avoid the problem of enlarging the aspect ratio of incorrectly predicted boxes in the GIoU loss function, thus improving the convergence ability of the network. Furthermore, our YOLOv5-MR model adds the detection of the $C_{2}$ feature layer, which contains more object information, and sets a group of smaller Anchors based on the target size characteristics of the MR-Meter dataset to speed up the convergence speed of predicted boxes for small targets, improving the model’s detection ability for small targets. Finally, the YOLOv5-MR model can learn better up-sampling parameters through transposed convolution, which helps the model converge faster and more stably.

### 4.5. Ablation Studies

To evaluate the effects of multiscale feature detection, EIoU loss function, and transposed convolution on object detection performance under the same experimental conditions, ablation experiments were conducted. The Ultralytics 5.0 version of the YOLOv5m model was used as the baseline model. The input image resolution was set to 640 × 640, and the model was trained for 300 epochs. The results are shown in Table 3. The second row of Table 3 shows that after introducing multiscale feature detection, the average precision increased by 1.2%, but the computation speed decreased. The third row of Table 3 shows that after using the EIoU loss function, the average precision increased by 1.8%, and the speed did not decrease. The fourth row of Table 3 shows that after replacing the nearest neighbor interpolation with transposed convolution, the average precision decreased by 0.3%. Due to unreasonable hyper-parameter settings, such as kernel size, stride, and padding of the transposed convolutional layer, adjustments need to be made based on the MR-Meter dataset. By incorporating these three improvements into the YOLOv5-MR model, the average precision improved by 3.0%, and the detection performance of small targets was significantly improved.

### 4.6. Comparative Experiment

In this section, we compared the performance of our proposed YOLOv5-MR model against several state-of-the-art object detection models, including Ultralytics5.0 version of YOLOv3 [20], YOLOv3-spp [35], and YOLOv5 [22]. The models were trained and validated using the MR-Meter dataset, and the comparison was conducted in terms of Recall, mAP, GFLOPS, and Weights, as presented in Table 4. It can be observed from Table 4 that our proposed YOLOv5-MR model achieved significantly higher mAP accuracy compared to YOLOv3, YOLOv3-spp, YOLOv5s, and YOLOv5m models.

Although the GFLOPS of our proposed model is lower than that of YOLOv5s and YOLOv5m models, it still satisfies the requirements of real-time and accuracy. Our YOLOv5-MR model introduced multi-scale feature detection and detected the $C_{2}$ feature layer with more object feature information to improve the extraction ability of small object features. Furthermore, we employed a better EIoU loss function that directly penalizes the side length to solve the problem of enlarging the wrong side length during the bounding box convergence process, which greatly improved the convergence speed of the model. Additionally, using transpose convolution in the backbone network can better learn the parameters of up-sampling and improve the accuracy of the model. This paper compares and analyzes the YOLOv5-MR algorithm with YOLOv7 [36] and YOLOv8 [37], and our findings indicate that our proposed algorithm exhibits excellent performance in terms of accuracy and recall. Specifically, compared to YOLOv7 and YOLOv8, our algorithm exhibits higher mAP and recall.

## 5. Disscussion

In this paper, we propose a new automated meter reading model, YOLOv5-MR. The YOLOv5-MR model has added a small object detection layer to predict small objects on the $\;C_{2}$ feature layer. In accordance with the characteristics of the MR-Meter dataset, a new set of anchors has been added to detect small objects, greatly improving the model’s ability to detect small objects. The YOLOv5-MR model directly uses the length of the predicted box as the penalty term, effectively avoiding the problem of amplifying the wrong length in the original IOU loss function, making the model converge quickly and well. Experimental results demonstrate that the YOLOv5-MR model outperforms the original YOLOv5 model in small object detection, and the new external circle fitting method is more accurate, faster, and more robust than current target-based meter reading methods. Compared to other recent meter reading studies, the YOLOv5-MR model achieves better results in accuracy, speed, and robustness. Compared to the recent single-stage YOLO network, our proposed algorithm shows excellent performance in accuracy and recall. Specifically, compared to YOLOv7 [36] and YOLOv8 [37], our algorithm shows higher mAP and recall rate.

## 6. Conclusions

This paper proposes an automatic meter reading model of dial indicator based on YOLOv5-MR, aiming at the problems of low accuracy and large error of current target-detection-based meter reading methods. The algorithm has a high accuracy and robustness. Through improving the multi-scale, Loss function and the way of using the target, the detection ability of the model is improved, and finally a new dial fitting circle algorithm is proposed. Accurate reading of the dial scale has been achieved. In the next phase, we intend to utilize a more lightweight backbone network and apply a model pruning algorithm to further reduce the weight of our model, thereby improving its overall efficiency. This will enable the model to operate with less computational resources and make it more suitable for use in resource-constrained settings.

Future research can explore how to apply this method to other fields while further improving the model’s performance and accuracy. The YOLOv5-MR model can be applied to fields such as substation automatic inspection robots and autonomous driving to improve their performance and accuracy.

## Figures and Tables

**Table 1:** Parameter settings.

Parameter Names	Parameter Values
Momentum	0.937
Weight_decay	0.0005
Batch_size	4
Learning_rate	0.01
Epochs	600

**Table 2:** The average error comparison of YOLOv5-MR and other object detection models.

Model	Average Error δ
YOLOv3	0.05
YOLOv3-SPP	0.048
YOLOv5s	0.046
YOLOv5m	0.028
YOLOv5-MR	0.022

**Table 3:** Ablation experiments.

Model	Recall (%)	mAP (%)	GFLOPS (s)	Weights (MB)
YOLOv5m	75.2	76.4	53.8	44.8
YOLOv5m + Multi-scale feature detection	76.2	77.6	94.6	49.0
YOLOv5m + EIoU Loss	76.6	78.2	53.8	44.8
YOLOv5m + Transposed Convolution	74.8	76.1	53.9	44.8
YOLOv5-MR	78.2	79.7	94.9	49.1

**Table 4:** Comparison of different object detection models.

Model	Recall (%)	mAP (%)	GFLOPS (s)	Weights (MB)
YOLOv3	74.6	75.0	158.1	125.4
YOLOv3-SPP	74.5	75.9	159.0	127.0
YOLOv5s	63.9	60.8	15.9	13.9
YOLOv5m	75.2	76.4	53.8	44.8
YOLOv7s	71.6	69.5	17.3	57
YOLOv8s	72.4	68.4	19.3	58
YOLOv5-MR	78.2	79.7	94.9	49.1

## Data Availability

Not applicable.
